# Epilepsy as a Comorbidity in Polymyositis and Dermatomyositis—A Cross-Sectional Study

**DOI:** 10.3390/ijerph18083983

**Published:** 2021-04-10

**Authors:** Ella Nissan, Abdulla Watad, Arnon D. Cohen, Kassem Sharif, Johnatan Nissan, Howard Amital, Ora Shovman, Nicola Luigi Bragazzi

**Affiliations:** 1 Department of Medicine ‘B’ & Zabludowicz Center for Autoimmune Diseases, Sheba Medical Center, Tel Hashomer 5262000, Israel; ella.nissan27@gmail.com (E.N.); watad.abdulla@gmail.com (A.W.); Kassemsharif@gmail.com (K.S.); Howard.Amital@sheba.health.gov.il (H.A.); orashovman@walla.co.il (O.S.); 2 Sackler Faculty of Medicine, Tel-Aviv University, Tel Aviv 69978, Israel; Johnatan.n@gmail.com; 3 Chief Physician’s Office, Clalit Health Services, Tel Aviv 16250, Israel; arcohen@clalit.org.il; 4 Siaal Research Center for Family Medicine and Primary Care, Faculty of Health Sciences, Ben-Gurion University of the Negev, Beer Sheva 8489325, Israel; 5 Department of Diagnostic Imaging, Sheba Medical Center, Tel Hashomer 5262000, Israel; 6 Laboratory for Industrial and Applied Mathematics (LIAM), Department of Mathematics and Statistics, York University, Toronto, ON M3J 1P3, Canada

**Keywords:** polymyositis, dermatomyositis, epilepsy, comorbidity, autoimmunity

## Abstract

Polymyositis (PM) and dermatomyositis (DM) are autoimmune-mediated multisystemic myopathies, characterized mainly by proximal muscle weakness. A connection between epilepsy and PM/DM has not been reported previously. Our study aim is to evaluate this association. A case–control study was conducted, enrolling a total of 12,278 patients with 2085 cases (17.0%) and 10,193 subjects in the control group (83.0%). Student’s t-test was used to evaluate continuous variables, while the chi-square test was applied for the distribution of categorical variables. Log-rank test, Kaplan–Meier curves and multivariate Cox proportional hazards method were performed for the analysis regarding survival. Of the studied 2085 cases, 1475 subjects (70.7%) were diagnosed with DM, and 610 patients (29.3%) with PM. Participants enrolled as cases had a significantly higher rate of epilepsy (*n* = 48 [2.3%]) as compared to controls (*n* = 141 [1.4%], *p* < 0.0005). Using multivariable logistic regression analysis, PM was found only to be significantly associated with epilepsy (OR 2.2 [95%CI 1.36 to 3.55], *p* = 0.0014), whereas a non-significant positive trend was noted in DM (OR 1.51 [95%CI 0.99 to 2.30], *p* = 0.0547). Our data suggest that PM is associated with a higher rate of epilepsy compared to controls. Physicians should be aware of this comorbidity in patients with immune-mediated myopathies.

## 1. Introduction

Polymyositis (PM) and dermatomyositis (DM) are immune-mediated myopathies with classical proximal muscle weakness, distinctive electromyography (EMG) abnormalities, elevated creatine kinase (CK) levels and inflammatory infiltration on muscle biopsy. Polymyositis and dermatomyositis are systemic, heterogenous diseases with significant morbidity, with proclivity to affect the skin, joints, gastrointestinal tract, and cardiovascular and respiratory systems. A diagnosis of DM is considered when characteristic rash is accompanied by myopathy detected by physical examination, histological findings, or EMG. For a diagnosis of PM, elevated CK levels accompanied by inflammatory infiltrate on muscle biopsy with subacute acquired clinical myopathy support the diagnosis [1,2].

It has been well established that immune-mediated myopathies (IIMs) are associated with several other conditions, including the strong link between malignancy and myositis, especially in the DM subset patients. These findings have been well described and replicated in several studies conducted across different populations. In most cases, cancer has been detected within the first year(s) after diagnosis [3,4,5,6].

The correlation between autoimmune/rheumatic disorders and neurologic disorders has also been widely investigated [7]. Of the common rheumatological disorders, both systemic lupus erythematosus (SLE) and rheumatoid arthritis (RA) have been frequently linked to higher rates of epilepsy [8,9,10].

Alas, the involvement of the central nervous system (CNS) in IIMs, especially in DM, has been poorly addressed before, instead reported as anecdotal and documented mainly by clinical reports and case series, with scant studies exploring the serum biomarkers potentially associated with both PM/DM and epilepsy [2,11,12,13,14,15,16,17,18,19,20].

To the best of our knowledge, an association between epilepsy and PM/DM has not been looked into and epidemiological studies from large population-level databases are much needed. Therefore, the aim of our study is to assess the possible association between PM/DM and epilepsy, the role of PM/DM-related autoantibodies and, finally, whether the association of PM/DM and epilepsy has any impact on patient survival rate.

## 2. Material and Methods

### 2.1. Ethical Statement

The study protocol was accepted by the Clalit Health Services (CHS), based at Soroka Medical Center, Beer-Sheva, Israel, and by the committee of ethics of Tel Aviv University, Tel Aviv, Israel. The ethical code number is 0212-17-COM2 and the date is 17 March 2020.

### 2.2. Study Design

The database was provided by the CHS’s chronic diseases registry, Israel’s largest health service with more than 4,400,000 insured (approximately 50% of the population in Israel) responsible for the health surveillance of approximately half of Israel population and one of the largest public health care organizations. The data was collected from 2000 to 2018, from different sources (medical, administrative and pharmaceutical) through a well-integrated computerized operating systems network. Massive data mining methods were performed to collect relevant information. Any participant diagnosed with PM/DM was selected, with each case being sex and age matched to approximately 5 controls selected from the database randomly. The present study is part of a series of population-based, large-scale studies intended to quantitatively assess the impact of co-morbidities on PM/DM patient outcomes.

### 2.3. Measures

To ensure reliability, PM/DM diagnosis was based on a documented diagnosis recorded in the medical documents at least twice (provided by either a certified primary care physician, a family doctor, or a specialist).

Similarly, a case of epilepsy was defined with at least two documented diagnoses in the medical records. In our study, the medical conditions corresponded to the following diagnostic codes -ICD-9 710.3 for DM, 710.4 for PM and 345 for epilepsy. Data present in the CHS database undergoes series of verification by comparing diagnoses from various sources. The validity of the data was confirmed to be high in previous studies [21].

Sociodemographic parameters such as age, gender, smoking status, socioeconomic status (SES), mortality and body mass index (BMI) were also extracted from the database. SES was calculated based on the 2008 Israeli National Census poverty index complying with the poverty index of the member’s residence area. Educational level, car ownership, household income and living conditions were among the several parameters used to compute such indices. The poverty index ranged from 1 to 20 (the lowest score and the highest score, respectively) which was then used to classify the study population into three main groups: low, medium, and high SES.

The sera of PM/DM subjects were collected routinely and examined for the presence of autoantibodies including anti-Sjögren’s syndrome-related antigen A (anti-SSA), antinuclear (ANA), anti-Sjögren’s syndrome-related antigen B (anti-SSB), anti-Jo-1 and anti-ribonucleoprotein (anti-RNP). Each test was interpreted using the respective cut-off values provided by the kit assay insert and manufacturers’ instructions. An assay was reported as either “positive” or “negative” using such thresholds. Had more than one samples been withdrawn from the subject, a “positive” result would have been reported provided at least one single positive result.

### 2.4. Statistical Analysis

Raw data was examined visually to eliminate potential outliers to ensure data distribution normality which was confirmed using the Pearson–D’Agostino omnibus test. Categorical variables were expressed as percentages and continuous variables were presented as means ± standard deviations.

Both univariate analyses and multivariable analyses were conducted to verify the hypothesis of the potential connection between PM/DM and epilepsy. The univariate analyses performed for categorical parameters were chi-squared test/Fisher’s test, whereas for continuous variables the methods used were analysis of variance (ANOVA), Student’s t-test or their non-parametric versions were performed in case of violation of normality of data distribution. Multivariable analyses consisted of logistic regressions. The variables used in the multivariate analyses were selected due to their known association with PM/DM or other autoimmune rheumatic disorders [22,23,24]

Survival analyses, using the log-rank test and a Kaplan–Meier curve for univariate and the Cox proportional hazards regression analysis for multivariate, were conducted to elucidate the impact of epilepsy as a co-morbidity on PM/DM subjects in terms of mortality.

All statistical analyses were conducted through the commercial software “Statistical Package for the Social Sciences” (SPSS version 24.0, IBM, Armonk, NY, USA). A Kaplan–Meier curve was conducted with the commercial software MedCalc (version 18.11.3, MedCalc Software bvba, Ostend, Belgium).

## 3. Results

A total of 12,278 subjects were included in our analysis, with 2085 PM/DM cases (17.0%) and 10,193 age- and gender-matched controls (83.0%). A total of 5042 were males (41.1%) and 7236 were females (58.9%), with a mean age of 47.81 ± 22.51 years. A total of 1475 patients (70.7%) were diagnosed with DM, and another 610 (29.3%) were diagnosed with PM. The average BMI of the study population was 26.89 ± 8.20 kg/m^2^ and 3695 of subjects (30.1%) were current smokers. One-hundred and eighty-nine patients (1.5%) had epilepsy and 466 patients (3.8%) died during the study period.

Basic characteristics between the case and control groups were similar, with no significant differences in terms of gender, age, SES distribution, BMI and smoking status. As a comorbidity, epilepsy was significantly more pronounced in the PM/DM cases in comparison to controls (2.3% vs. 1.4%;, respectively, *p* < 0.0005). The all-cause mortality rate was significantly higher among the PM/DM cohort (7.6% vs. 3.0%, *p* < 0.0001), with 115 deaths demonstrated in the DM subset (7.8%) and another 44 deaths among PM subjects (7.2%) (shown in Table 1).

Using the multivariable logistic regression model, PM was significantly associated with epilepsy (OR 2.2 [95%CI 1.36–3.55], *p* = 0.0014). In comparison, a similar trend of association with epilepsy was noted in the DM subset, but not crossing the significance threshold (OR 1.51 [95%CI 0.99 to 2.30], *p* = 0.0547). When combined, PM/DM was associated with increased risk of epilepsy (OR 1.74 [95%CI 1.24 to 2.44], *p* = 0.0013). All the other variables that were evaluated did not significantly contribute to the risk of developing epilepsy (shown in Table 2).

**Figure 1:**
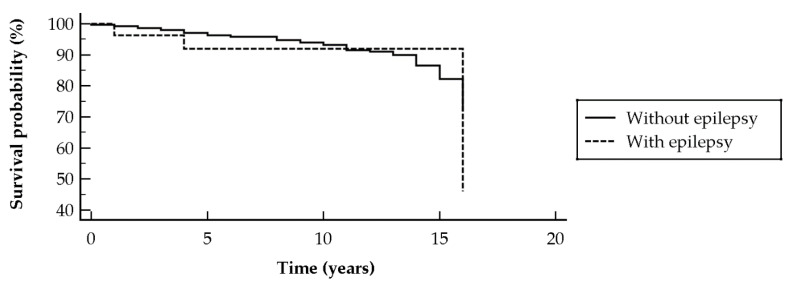
Kaplan–Meier survival curve of dermatomyositis patients with and without epilepsy, showing no significant impact on mortality rate.

**Figure 2:**
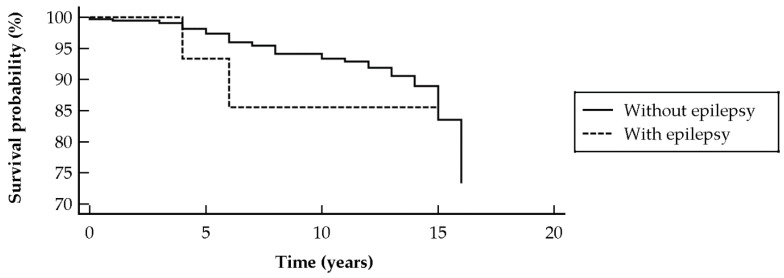
Kaplan–Meier curve survival of polymyositis patients with and without epilepsy, showing no significant impact on mortality rate.

There was no impact of a diagnosis of epilepsy on the survival probability of DM subjects (chi-squared = 0.22, *p* = 0.6365; hazard ratio (HR) 0.73 [95% CI 0.19–2.73]) (shown in Figure 1), and PM patient survival rate (chi-squared = 0.56, *p* = 0.4536; HR 0.50 [95% CI 0.08–3.10]) (shown in Figure 2).

PM subjects were positive for autoantibodies ANA, anti-Jo1, anti-SSA, anti-SSB and anti-RNP in 42.8%, 21.0%, 30.5%, 27.2% and 6.3% of the cases, respectively. Similarly, DM patients were positive for ANA (32.5%), anti-Jo1 (16.1%), anti-SSA (30.5%), anti-SSB (28.0%), and anti-RNP (9.4%). Using multivariable logistic regression analyses, autoantibodies were not significantly associated with epilepsy among PM/DM subjects (shown in Table 3).

## 4. Discussion

We reported in our study an association between PM/DM and epilepsy and, to the best of our knowledge, our cross-sectional study is the first to investigate such a relationship from a large population-based database.

Autoimmune/rheumatic disorders have a well-documented link to CNS involvement, plausibly explained by the concurrent inflammatory process affecting the brain, or the role of anti-rheumatic therapies employed to treat the underlying disease. In some studies, RA and SLE have been well associated with a high rate of CNS manifestations, including epilepsy [7,8,9,10].

In the current literature, the association between immune-mediated myopathies and CNS disorders has not been well investigated and attracted minimal attention, with only a few clinical reports and cases series delineating a possible association [11,12,25,26,27].

While the literature has overlooked the connection between PM/DM and epilepsy, several case reports have proposed a possible link between anti-epileptic drugs (phenytoin and gabapentin) and the development of PM/DM. One case study suggested the role of gabapentin, namely by the modulation of calcium channels in skeletal myocytes, in the development of gabapentin-induced myositis [28,29,30].

Moreover, both epilepsy and PM/DM share a putative infectious etiology which could go on to explain such an association. Infections are among the most prevalent risk factor for acquired epilepsy, mainly involving the CNS, while on the other hand, infectious myositis may result from immune-mediated mechanisms without directly infecting the muscle tissue. Further, the treatment of PM/DM patients with immunosuppressive agents could, in itself, predispose patients to infectious diseases which would result in epilepsy. Studies have shown that several infectious agents such as bacteria, fungi, parasites, and viruses could lead to autoimmune/rheumatologic conditions but a plausible mechanism underlying this association is still to be elucidated [31,32,33].

In our study, a significant association between PM/DM and epilepsy was documented. Although not reaching statistical significance, the risk of epilepsy trended positively with a diagnosis of DM. Another caveat in the current literature is the lack of association studies between PM/DM and epilepsy biomarkers. Both have an inflammatory infiltrate in the respective tissue (brain after epileptic seizures and muscle in PM/DM) with shared cytokine over-expression including IL-1β, IL-6 and TNFα [2,13]. Studies about serum immune biomarkers in epileptic patients are mainly evaluated after an epileptic seizure and thus comparison to PM/DM is limited. Moreover, no studies are underway to examine a biomarker commonly shared by both disorders, although a group of studies report a shared high concentration of IL-6, TNFα, and Fas in the blood sera of patients [13,14,15,16,17]. Other studies investigated non-immune serum biomarkers—for instance, CK was studied and found to be independently associated with PM/DM or seizures separately, but no studies evaluated the interaction and elevation of CK in a cohort of PM/DM patients with epilepsy as a comorbidity [2,18,19]. Furthermore, seizures caused by autoimmune and paraneoplastic neurological disorders have been linked to autoantibodies [34,35], none of which match with those prevalent in PM/DM [20].

Of note, the recent COVID-19 pandemic uncovered possible therapeutic approaches for refractory epilepsy. This form of epilepsy developing secondary to COVID-19 is suggested to have an autoimmune etiology and a good response to intravenous immunoglobulin treatment was documented [36]. In terms of serum autoantibodies, we could not identify a statistically significant association between any of the common PM/DM autoantibodies and epilepsy in PM/DM patients. However, we cannot rule out that the association between PM/DM and epilepsy may be related to the presence of other autoantibodies that were not included in the routine diagnostic work up of our patients. For instance, epilepsy is present in 10% of patients with antiphospholipid antibodies [37] and such antibodies might also be present in PM/DM patients [38].

Although epilepsy represents a high-risk factor for all-cause mortality in the general population [39,40], epilepsy did not result in increased mortality in PM/DM patients in our study. Our study has several limitations that warrant consideration. The observational design and lack of temporal relationship precludes the determination of a causal relationship between PM/DM and epilepsy. Moreover, input regarding medications used and disease period was not available. However, these limitations are supplanted by the strengths, including the use of data in a real-life setting, thus mirroring existing currents in the population.

## 5. Conclusions

Our study reports a significant association between PM and epilepsy. Physicians should take into consideration this epidemiologic association while treating PM/DM patients with such comorbidity. More studies are required to further elucidate the mechanisms underpinning this association.

## Figures and Tables

**Table 1:** Main population characteristics recruited in the present study.

Parameters	Entire Population (*n* = 12,278)	Controls (*n* = 10,193)	Cases (*n* = 2085)			Significance
			Dermatomyositis/Polymyositis (*n* = 2085)	Dermatomyositis (*n* = 1475)	Polymyositis (*n* = 610)	
Age	47.81 ± 22.51	47.75 ± 22.50	48.10 ± 22.56	43.68 ± 22.79	58.79 ± 17.97	NS
Gender						NS
Male	5042 (41.1%)	4186 (41.1%)	856 (41.1%)	639 (43.3%)	217 (35.6%)	
Female	7236 (58.9%)	6007 (58.9%)	1229 (58.9%)	836 (56.7%)	393 (64.4%)	
BMI	26.89 ± 8.20	26.93 ± 8.58	26.73 ± 6.07	26.19 ± 6.03	27.88 ± 6.01	NS
SES						NS
Low	5147 (42.1%)	4277 (42.2%)	870 (41.9%)	614 (41.9%)	256 (42.2%)	
Medium	4454 (36.5%)	3694 (36.4%)	760 (36.6%)	524 (35.7%)	236 (38.9%)	
High	2616 (21.4%)	2172 (21.4%)	444 (21.4%)	329 (22.4%)	115 (18.9%)	
Smoking	3695 (30.1%)	3079 (30.2%)	616 (29.5%)	402 (27.3%)	214 (35.1%)	NS
Epilepsy	189 (1.5%)	141 (1.4%)	48 (2.3%)	28 (1.9%)	20 (3.3%)	0.0005
All-cause mortality rate	466 (3.8%)	307 (3.0%)	159 (7.6%)	115 (7.8%)	44 (7.2%)	<0.0001

BMI, body mass index; CI, confidence interval; NS, not significant; SES, socioeconomic status.

**Table 2:** Multivariable logistic regression analysis investigating co-variates associated with epilepsy.

Variable	Coefficient	Standard Error	Wald	*p*-Value	Odds Ratio	95% CI
Age	0.01	0.00	5.67	0.0173	1.01	1.00 to 1.02
Female vs. male	−0.11	0.16	0.43	0.5137	0.90	0.66 to 1.23
BMI	0.00	0.01	0.02	0.8916	1.00	0.98 to 1.02
Current smoker	−0.02	0.17	0.01	0.9205	0.98	0.71 to 1.36
SES						
Medium	−0.03	0.17	0.04	0.8482	0.97	0.69 to 1.35
High	−0.25	0.21	1.40	0.2374	0.78	0.51 to 1.18
Dermatomyositis	0.41	0.21	3.69	0.0547	1.51	0.99 to 2.30
Polymyositis	0.79	0.25	10.26	0.0014	2.20	1.36 to 3.55
PM/DM	0.55	0.17	10.36	0.0013	1.74	1.24 to 2.44
Constant	−4.60	0.33	192.89	<0.0001		

BMI, body mass index; CI, confidence interval; DM, dermatomyositis; PM, polymyositis; SES, socioeconomic status.

**Table 3:** Multivariable logistic regression analysis examining the association between autoantibodies and the risk of developing epilepsy for PM/DM overall and stratified according to dermatomyositis and polymyositis.

Variable	Coefficient	Standard Error	Wald	*p*-Value	Odds Ratio	95% CI
**Anti-SSA**						
PM/DM	−0.28	0.52	0.29	0.5929	0.76	0.28 to 2.09
Dermatomyositis	0.58	1.02	0.33	0.5672	1.79	0.24 to 13.13
Polymyositis	1.39	1.25	1.25	0.2639	4.03	0.35 to 46.49
**Anti-SSB**						
PM/DM	18.58	7396.16	0.00	0.9980	0.00	
Dermatomyositis	17.56	7079.91	0.00	0.9980	0.00	
Polymyositis	17.59	6104.15	0.00	0.9977	0.00	
**ANA**						
PM/DM	0.27	0.36	0.54	0.4628	1.30	0.64 to 2.65
Dermatomyositis	−0.01	0.51	0.00	0.9899	0.99	0.37 to 2.68
Polymyositis	0.51	0.56	0.82	0.3648	1.66	0.56 to 4.94
**Anti-Jo1**						
PM/DM	0.15	0.57	0.07	0.7962	1.16	0.38 to 3.57
Dermatomyositis	−19.90	9318.37	0.00	0.9983	0.00	
Polymyositis	0.84	0.65	1.66	0.1972	2.31	0.65 to 8.20
**Anti-RNP**						
PM/DM	−19.65	8059.58	0.00	0.9981	0.00	
Dermatomyositis	−18.78	6255.66	0.00	0.9976	0.00	
Polymyositis	−18.38	7951.13	0.00	0.9982	0.00	

Anti-SSA, anti-Sjögren’s syndrome-related antigen A; ANA, antinuclear antibody; anti-RNP, anti-ribonucleoprotein; anti-SSB, anti-Sjögren’s syndrome-related antigen B; PM, polymyositis; DM, dermatomyositis; CI, confidence interval.

## Data Availability

The datasets generated during and/or analysed during the current study are not publicly available due to the local ethics committee decision, but are available from the corresponding author on reasonable request.

## Abbreviations

DM	dermatomyositis
PM	polymyositis
ANA	antinuclear antibody;
Anti-SSA	anti-Sjögren’s syndrome-related antigen A
Anti-SSB	anti-Sjögren’s syndrome-related antigen B
Anti-RNP	anti-ribonucleoprotein
BMI	body mass index
CK	creatine kinase
SLE	systemic lupus erythematosus
SES	socioeconomic status
RA	rheumatoid arthritis
